# Adipose-derived Stem Cells Attenuates Diabetic Osteoarthritis via Inhibition of Glycation-mediated Inflammatory Cascade

**DOI:** 10.14336/AD.2018.0616

**Published:** 2019-06-01

**Authors:** Navneet Kumar Dubey, Hong-Jian Wei, Sung-Hsun Yu, David F. Williams, Joseph R. Wang, Yue-Hua Deng, Feng-Chou Tsai, Peter D. Wang, Win-Ping Deng

**Affiliations:** ^1^Graduate Institute of Biomedical Materials and Tissue Engineering, College of Biomedical Engineering, Taipei Medical University, Taipei, Taiwan; ^2^Stem Cell Research Center, College of Oral Medicine, Taipei Medical University, Taipei, Taiwan; ^3^School of Dental Technology, College of Oral Medicine, Taipei Medical University, Taipei, Taiwan; ^4^School of Dentistry, College of Oral Medicine, Taipei Medical University, Taipei, Taiwan; ^5^Wake Forest Institute of Regenerative Medicine, Winston-Salem, NC, USA; ^6^Department of Periodontics, College of Dental Medicine, Columbia University, New York, USA; ^7^Department of Life Science, Fu Jen Catholic University, New Taipei City, Taiwan; ^8^Stem Cell Research Center, Cosmetic Clinic Group, Taipei, Taiwan; ^9^Department of Dentistry, Taipei Medical University Hospital, Taipei, Taiwan; ^10^Graduate Institute of Basic Medicine, Fu Jen Catholic University, New Taipei City, Taiwan

**Keywords:** Diabetes mellitus, osteoarthritis, articular cartilage, proteoglycans, advanced glycation end product, adipose-derived stem cells

## Abstract

Diabetes mellitus (DM) is well-known to exert complications such as retinopathy, cardiomyopathy and neuropathy. However, in recent years, an elevated osteoarthritis (OA) complaints among diabetics have been observed, portending the risk of diabetic OA. Since formation of advanced glycation end products (AGE) is believed to be the etiology of various diseases under hyperglycemic conditions, we firstly established that streptozotocin-induced DM could potentiate the development of OA in C57BL/6J mouse model, and further explored the intra-articularly administered adipose-derived stem cell (ADSC) therapy focusing on underlying AGE-associated mechanism. Our results demonstrated that hyperglycemic mice exhibited OA-like structural impairments including a proteoglycan loss and articular cartilage fibrillations in knee joint. Highly expressed levels of carboxymethyl lysine (CML), an AGE and their receptors (RAGE), which are hallmarks of hyperglycemic microenvironment were manifested. The elevated oxidative stress in diabetic OA knee-joint was revealed through increased levels of malondialdehyde (MDA). Further, oxidative stress-activated nuclear factor kappa B (NF-κB), the marker of proinflammatory signalling pathway was also accrued; and levels of matrix metalloproteinase-1 and 13 were upregulated. However, ADSC treatment attenuated all OA-like changes by 4 weeks, and dampened levels of CML, RAGE, MDA, NF-κB, MMP-1 and 13. These results suggest that during repair and regeneration, ADSCs inhibited glycation-mediated inflammatory cascade and rejuvenated cartilaginous tissue, thereby promoting knee-joint integrity in diabetic milieu.

Diabetes mellitus (DM), a non-communicable disorder, currently constitutes a major epidemic, affecting children, adolescents and adults, ultimately resulting in several disastrous complications [1, 2]. However, the nature of any association between DM and OA remains unclear. Particularly, it is uncertain whether DM predisposes to knee osteoarthritis (OA), which is typically characterized by deteriorative changes in the osteochondral unit, comprising of cartilage (hyaline and calcified), meniscus (fibrocartilage) and subchondral bone [3]. So far, only epidemiological studies have explored this possibility, and according to a report by Louati et al., a mean prevalence of OA among DM patients is 29.5±1.2%, whereas a mean prevalence of DM among OA patients has been reported as 14.4±0.1% [4]. The underlying mechanisms of this relationship have not been identified, and there is no adequate evidence for a conclusive model of DM-induced OA. The connecting link between these two disorders may be supported by the deleterious role of elevated blood glucose levels in DM through the accumulation of advanced glycation end products (AGEs) in various tissues. AGEs are a variety of chemically modified proteins, such as N^ε^-(carboxymethyl)-lysine (CML), pyrraline, pentosidine, or other cross-linked molecules [5], and are implicated in various pathologies. However, the impact of AGEs has not yet been explored in osteoarthritic knee-joints. Furthermore, the receptor for AGE (RAGE) is the best-characterized cell surface molecule to which AGEs bind, and it has been implicated in many cell types [6, 7]. The formation of AGEs and their interaction with RAGE activate intracellular signalling pathways causing cellular oxidative stress via production of reactive oxygen species, which further activate nuclear factor κB (NF-κB), leading to transcription of pro-inflammatory genes [8, 9]. Hence, the AGE-RAGE axis mediated inflammatory response might be potential therapeutic target for diabetic OA.

Traditional therapeutic strategies for managing OA have proven their efficacy solely in relieving pain, however, exert side effects and are unable to reverse cartilage damage; of these, a few reported cellular therapies have provided the hope for the future [10]. The major concern of diabetic complications is that the tissue healing remains delayed in hyperglycemic environment due to functional deterioration of resident and recruited cells [11]. To date, therapies that potentially address such complications are limited to drugs, which mostly aim at controlling the blood glucose levels [12]. To our knowledge, no treatment has been investigated for reversing the hyperglycemia-induced osteoarthritic changes. Thus, the development of novel regenerative strategies is urgently needed for structural and functional recovery in diabetic OA.

According to previous reports, mesenchymal stem cells (MSCs), particularly adipose-derived stem cells (ADSCs), have therapeutic potential due to their ease of isolation, enhanced proliferation, differentiation ability to various lineage, secretion of anti-inflammatory and anti-apoptotic molecules, and growth factors that support immunomodulation and protection from cellular apoptosis [13, 14]. During cell therapy, the two crucial factors, including cellular proliferation and their commitment to specific lineages play a significant role in rejuvenation of damaged tissue [15]. These regenerative efficacies might be attributed to anti-catabolic secretome of MSC during their phases of differentiation [16, 17]. In this study, we firstly established streptozotocin (STZ)-induced DM in mice to investigate whether hyperglycemia can trigger OA-like phenotype. STZ, a toxic nitrosourea analogue, is most widely used diabetogenic agent for the generation of type 1 diabetes via the destruction of insulin-producing cells in mice [18]. Due to the higher sensitivity towards STZ, we employed C57BL/6J male mice as a diabetic OA model [19], in which a cellular therapeutic strategy using ADSCs has been explored.

## MATERIALS AND METHODS

### Experimental induction of diabetes in mice

7-week old male C57BL/6J mice were purchased from National Laboratory Animal Center, Taipei, Taiwan and were kept at the Laboratory Animal Center, Taipei Medical University (TMU). All the animal care and used protocols were as per guidelines of TMU Institutional Animal Care and Use Committee (IACUC) and prior approval was obtained by IACUC to conduct this study. Mice were acclimatized to the laboratory conditions for 2 weeks prior to the inception of experiments. Mice were fed with normal chow (LabDiet 5010). They were induced with DM via a single intraperitoneal injection of 200 mg/kg streptozotocin (STZ: S0130, Sigma-Aldrich, USA) freshly prepared in citrate buffer (pH 4.5). At 4 weeks after STZ injection, the mice were administered 1.0×10^6^ ADSC/knee-joint, or PBS, while controls received no any treatment.

### Assessment of blood glucose level

To corroborate the induction of DM, blood samples were collected from the tail-vein of STZ administered mice, and fasting blood glucose (FBG) was measured by glucose oxidase strips (Easytouch, Taiwan). The mice with blood glucose level exceeding 250 mg/dl were considered diabetic [23]. After 4 weeks, hyperglycemic mice which exhibited the blood glucose concentration in the range of 300-350 mg/dL were selected for further experiments associated with OA, these mice being designated as the diabetic osteoarthritis (DM-OA) group. This DM-OA group was then divided into two sub-groups. One was treated with PBS (DM-OA+PBS, n = 6), and the other group was treated with ADSC (DM-OA+ADSC, n = 6). The control group received no any treatment (Control, n = 5).

### Preparation and characterization of ADSC from mice

Inguinal fat pad adipose tissues were harvested from 8-week-old mice. For routine cultures, ADSC were maintained in α-MEM supplemented with 20% fetal bovine serum (FBS; Hyclone, Logan, UT) in a humidified atmosphere containing 5% CO_2_. Finally, the ADSC (passage 1 ~ 3) were selected for further experiments.

The immunophenotypes of ADSCs were determined by flow cytometry-based evaluations. The cells were trypsinized, washed, and resuspended in PBS at a density of 10^6^ cells/ml. After fixation, cells were washed twice, and cell pellets were resuspended in 0.5ml PBS containing primary antibody for 30 minutes. Cells were immunolabeled with the following mouse surface antigen-specific antibodies: CD105 (eBioscience, San Diego, CA), CD90, CD34, and CD45 (MACS, Bergisch Gladbach, Germany). The non-specific mouse IgG (eBioscience, San Diego, CA) was substituted for the primary antibodies as isotype control. Thereafter, cells were washed twice and resuspended in 0.5ml for flow cytometric analysis (Becton, Dickson and company, San Jose, CA).

For determining the multi-lineage differentiation potential, ADSC were cultured until reaching 90% confluence. For osteogenic differentiation, cells were cultured in α-MEM supplemented with 10% FBS, 0.1 μM dexamethasone (Sigma), 5 mM β-glycerophosphate (Sigma), and 50 μM ascorbic acid (Sigma) for 12 days. For adipogenic differentiation, cells were cultured in α-MEM supplemented with 10% FBS, 1M dexamethasone (Sigma), 0.5 mM isobutyl-methylxanthine (Sigma), 10 μg/ml insulin (Gibco BRL, Carlsbad, CA), and 100 μg/ml indomethacin (Sigma) for 12 days. For chondrogenic differentiation, the cells were cultured in α-MEM supplemented with 10% FBS and 10 ng/ml TGF-β1 (PeproTech, Rocky Hill, NJ) for 21 days. After culturing the ADSCs in the specific induction medium, their differentiation into osteocytes, adipocytes and chondrocytes were corroborated via staining with alizarin Red S, oil-red O, and alcian blue, respectively.

### ADSC labelling and bio-distribution of intra-articularly administered ADSC

ADSCs were fluorescently labelled with the nontoxic dye, 4μM chloro-methyl-benzamido-1,1’-dioctadecyl-3,3,3’3’-tetra-methyl-indo-carbocyanine per-chlorate (CM-DiL) (Molecular Probes, C7001) to enable long-term in vivo cell tracking. Specifically, a 4μM solution of CM-DiL in DMSO was added to 1×10^6^ ADSC and incubated for 15 minutes in a humidified CO_2_ incubator at 37°C and then incubated for an additional 15 minutes at 4°C prior to being pelleted, washed, and resuspended in PBS. After 4-week of DM induction, 10 μl of PBS containing 1×10^6^ CM-DiL labelled ADSC (ADSC+CM-DiL) were intra-articularly injected via 0.5-ml monoject (29-gauge) insulin syringe (BD Micro-Fine, USA) into the knee joint. The uptake of CM-DiL by ADSCs was monitored *in vitro* by fluorescence microscopy as well as laser confocal scanning (Leica TCS SP5, Leica Microsystems). The *in vivo* bio-distribution of administered ADSC+CM-DiL was evaluated at 2 and 4 weeks.

### Histology and immunohistochemical analysis

Mice were sacrificed after 4-week treatment of ADSC to DM-OA group mice (n=6 animals/group). Knee joint samples were harvested and fixed in neutral formalin for 2 days and decalcified in a rapid decalcifier (Nihon Shiyaku Industries Ltd., Osaka, Japan) for further sectioning. Decalcified samples were embedded in paraffin, and the samples were sectioned at 5 μm thickness along the sagittal plane. Slides were stained with hematoxylin and eosin (H&E) for determination of tissue architecture of articular cartilage. Safranin O staining and fast green (Sigma) staining was conducted to estimate the distribution of proteoglycans and OA grade was assessed using the OARSI scoring system [20]. Immunohistochemical (IHC) staining was done using the standard avidin-biotin-peroxidase complex technique. Tissue sections were then visualized using Vectastain Kit (Vector Laboratories). The images were obtained under 100X and 200X magnification, representing femoral and tibial regions in articular cartilage, and quantification was performed using ImageJ (NIH, Bethesda, MD).

### Immunoblotting

The dissected knee-joints of all the groups were pulverized to fine powder under liquid nitrogen using a tissue gun, placed in lysis radioimmunoprecipitation assay (RIPA) buffer (50 mM-Tris, 150 mM NaCl, 0.5% DOC, 1% NP-40, and 0.1% SDS) and sonicated at 4°C. The homogenates were centrifuged at 12,000 rpm for 30 min, and supernatants containing total protein were retained. The extracted proteins were denatured for 5 min at 95 °C and were loaded on 10% SDS-PAGE gel, which were were transferred on to the PVDF membrane, and blocked in 4% BSA blocking-buffer. The membrane was then reacted with primary antibodies. Membranes were then incubated with anti-rabbit secondary peroxidase-conjugated antibody (Cell Signaling, 7074P2). In addition, monoclonal antibodies were then incubated with anti-mouse secondary peroxidase-conjugated antibody (GeneTex, GTX213111-01). Bands were visualized by Hyperfilm (Amersham Pharmacia) using the ECL plus-kit (Millipore Corporation) and images were analyzed using Mutigel-21. Data were presented with reference to control intensities of β-actin. The antibodies employed were Col II (Abcam, ab34712), CML (Abcam, ab125145), RAGE (Abcam, ab3611), MDA (Abcam, ab6463), NF-κB (cell Signaling, #8242), MMP-13 (Abcam, 39012), AGN (Millipore, MABT83), MMP-1 (GeneTex, GTX100534) and β-actin (GeneTex, GTX109639).

### Statistical analysis

Data were expressed as mean ± standard error of mean (SEM). Results were analyzed using s*tudent’s t-test* and a p-value of <0.05 was considered statistically significant.

**Figure 1.**
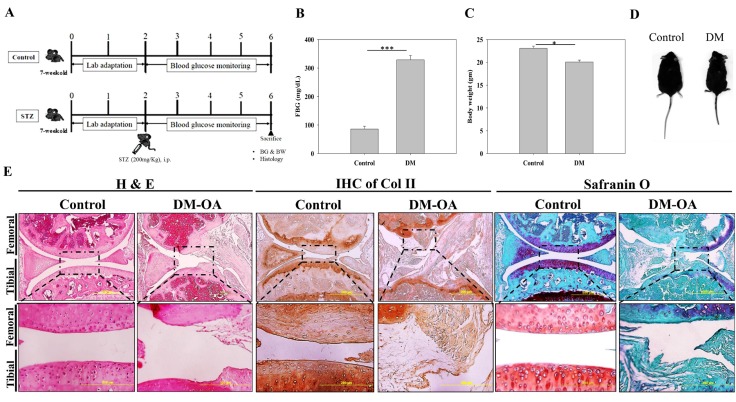
Establishment of diabetes mellitus (DM)-induced osteoarthritis (OA) mice (**A**) Schematic of experimental design representing establishment of streptozotocin (STZ)-induced DM. Mice at the age of 9 weeks were treated with single intraperitoneal injection of STZ (200mg/Kg body weight) or without STZ, designated as DM and control group, respectively. Measurement of fasting blood glucose (FBG) (**B**) and Body weight (**C**) of control and DM group mice after 4 weeks of STZ injection. (**D**) Representative gross appearance of control and DM mice showing distinct size differences. (**E**) Histologic assessment of OA characteristics in knee-joint of diabetic mice. The tissue sections were subjected to hematoxylin & eosin (H&E) staining, immunohistochemical staining of Col II (IHC Col II) and safranin O staining to determine the structural impairments, collagen and proteoglycan content, respectively in control and diabetes-induced osteoarthritis (DM-OA) group. The images in the upper panel are at lower magnification (scale bar: 500µm) in which the dotted rectangular boxed regions are represented as their respective images at higher magnification (scale bar: 200µm) in the lower panel. Data are shown as mean±SEM (Control, n=5; DM-OA, n=5). * p< 0.05 and *** p < 0.001.

## RESULTS

### Characterization of DM-induced OA

To establish DM-induced OA model, the C57/BL6 mice were firstly rendered diabetic via intraperitoneal injection of diabetogenic agent, streptozotocin (STZ; 200mg/Kg) and designated as DM-OA group; while healthy mice served as a non-diabetic control group (Fig. 1A, lower and upper panel, respectively). At third week, DM mice began to exhibit diabetic characteristics (blood glucose>180 mg/dL). By the end of 3-4 week, mice demonstrated sustained hyperglycemia compared to those of control. In particular, the fasting blood glucose (FBG) in the diabetic group was over 300 mg/dL (Fig. 1B) with a significant decrease in body weight (Fig. 1C) compared to control group. The DM mice appeared listless and lean compared to those of control (Fig. 1D). To examine the effect of diabetes on ultrastructural pathologic alterations associated with OA, the H&E staining of knee joint sections was performed (Fig. 1E, H&E panel). After careful examination of tissue sections, we found that control group revealed normal appearing articular cartilage in which the uppermost superficial acellular layer containing dense collagen fibres appeared intact and smooth. Many round, smaller and flattened (spindle-shaped) chondrocytes housed in lacuna were present, predominantly parallel to articular surface. Moreover, isogenous chondrocytes were surrounded by tangentially arranged collagenous fibres. The territorial and inter-territorial matrices were well-organized and more representative of healthy articular cartilage. The diabetic group (DM-OA) showed a complete loss of a superficial zone. Extremely loosened plexus of wavy collagen fibrils with an absolute loss of chondrocytes were observed, with no zonation. Invagination extending vertically deeper from articular surface to subchondral bone and fibrillation were also noticed. Overall, the control group did not exhibit any sign of structural damage, while a massive deterioration was evident in DM-OA group.

To further evaluate whether diabetes induced cartilage degradation, the immunohistochemical (IHC) staining of type II collagen was conducted. A heavily eroded and denatured type II collagen was exhibited in the DM-OA group, whereas control knee joints revealed a non-damaged appearance, with an intense focal staining in superficial and deeper regions (Fig. 1E, IHC-col II panel). Furthermore, to assess the influence of diabetes on aggrecan, a major proteoglycan in articular cartilage, the sections were evaluated by safranin o staining. The intense red signals indicated an abundant proteoglycan in the control group, while a massive degradation of proteoglycan in DM-OA group was found, suggesting OA-like changes in knee joint (Fig. 1E, IHC-aggrecan panel). These results indicated that hyperglycemic microenvironment participates in the pathology of OA in diabetes.

**Figure 2.**
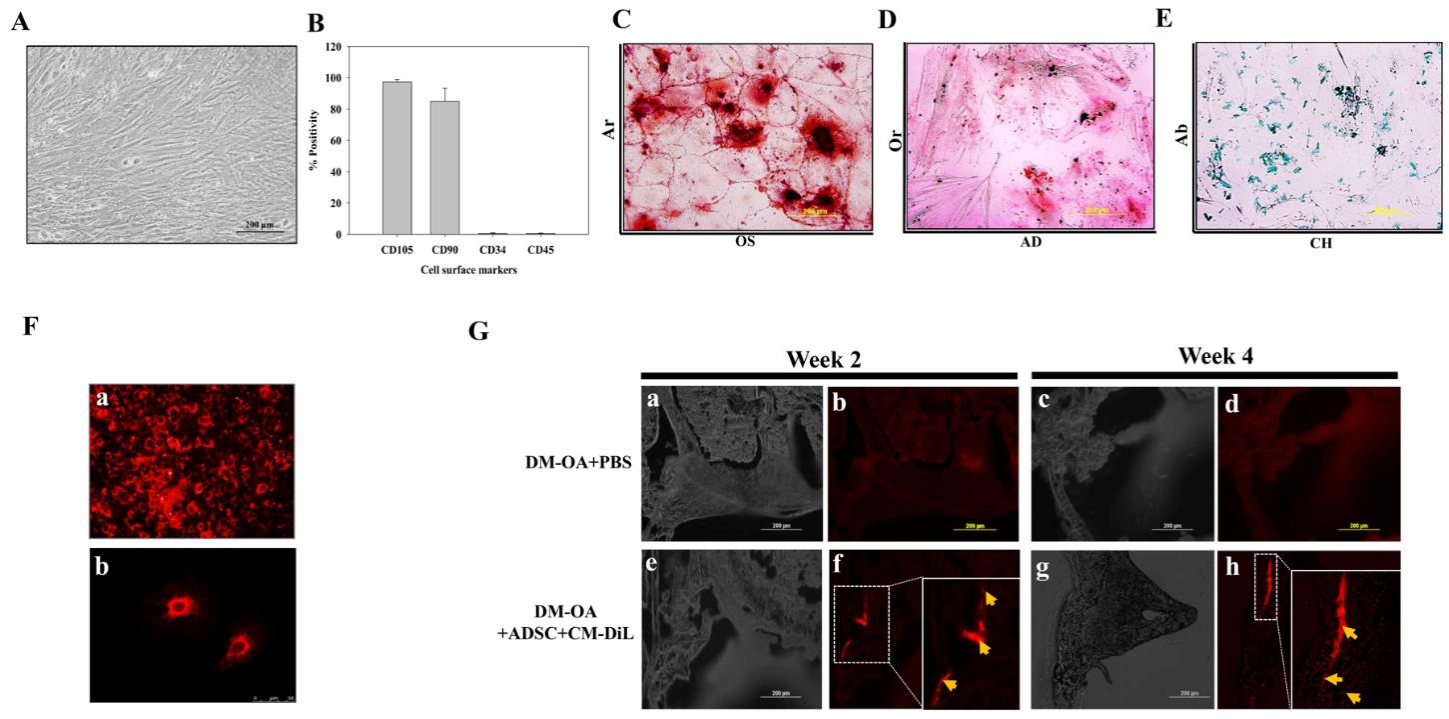
Characterization and corroboration of intra-articularly administered adipose-derived stem cells (ADSC) in knee joint of diabetic mice ADSC were harvested from mice and characterized for presence of mesenchymal stem cell properties. (**A**) Representative photomicrographs of morphological characteristics of ADSC. Scale bars: 200 µm, (**B**) Immunophenotype assessment of ADSC via flow cytometry assay of percentage of positives mesenchymal stem cells markers (CD105 and CD90) and negative hematopoietic markers (CD34 and CD45). Representative photomicrographs of ADSC in (**C**) osteogenic medium (OS) and further stained with alizarin red (Ar) dye, and in (**D**) adipogenic medium (AD) which was further stained with oil red (Or) dye, and in (**E**) Chondrogenic medium (CH) which was further stained with alcian blue (Ab) dye. Scale bar: 200 μm. Determination of *in vitro* uptake of chloromethylbenzamido dialkylcarbocyanine (CM-DiL) in ADSC (Scale bar: 200µm) and their further confirmation through (**F-a**) fluorescence microscope, Scale bar: 200µm, and (**F-b**) Confocal laser scanning microscopy, Scale bar: 50µm. (**G**) Detection of intra-articularly transplanted ADSC. *In vivo* homing of injected CM-DiL-stained ADSC (thick yellow arrows) in synovial areas of knee-joint at week 2 and 4 (lower panel, f and h), respectively. Scale bars: 200µm.

### Intra-articular incorporation of injected ADSCs

Prior to administration of cellular therapy, ADSC (P1-P3) from mice were isolated and characterized. The morphological characteristics were observed via inverted phase-contrast microscopy in which the ADSCs appeared as a normal, elongated and fibroblast-like phenotype, (Fig. 2A) and displayed a high capacity to adhere to the plastic disc. Next, to assess the immunophenotype of ADSCs, we performed flow cytometric analyses of cell surface markers profiles. The results indicated that the ADSCs demonstrated almost similar profiles that are characteristic to MSCs (positive for CD105, CD90 and; negative for CD34, CD45) (Fig. 2B). Thereafter, we evaluated the differentiation potential of ADSCs towards osteogenic, adipogenic, and chondrogenic lineages. Upon osteogenesis (OS), the ADSC group showed calcium deposits stained as many dark red regions (Fig. 2C). Upon adipogenesis (AD), ADSCs displayed the formation of lipid droplets which positively stained red (Fig. 2D). Lastly, chondrogenesis (CH) was confirmed by formation of dark blue proteoglycan-producing pellets by using alcian blue staining (Fig. 2E). The above results indicated that isolated ADSCs preserved the MSC characteristics.

In order to detect the internalization of intra-articularly administered ADSCs into knee joints of diabetic mice, we confirmed the *in vitro* uptake of CM-DiL dye into ADSCs. Following the cell labelling and their subsequent culture, an intense red coloured signal of ADSC+CM-DiL under fluorescent microscope (Fig. 2F-a) and confocal laser microscope (Fig. 2F-b) were observed, which corroborated the uptake of CM-DiL. Thereafter, intra-articularly administered CM-DiL labelled ADSC were visualized in the knee-joint (Fig. 2G). Two and four weeks post-injection of cells, knee-joint were harvested, and the histologic analysis revealed highly positive CM-DiL red signals in synovial regions, indicating the internalization of ADSC and their homing in reparative tissues. Notably, compared to 2 weeks (Fig. 2G-f), the enhanced CM-DiL positive signals at 4 weeks (Fig. 2G-h) indicated that administered ADSC increased rapidly in number over time.

**Figure 3.**
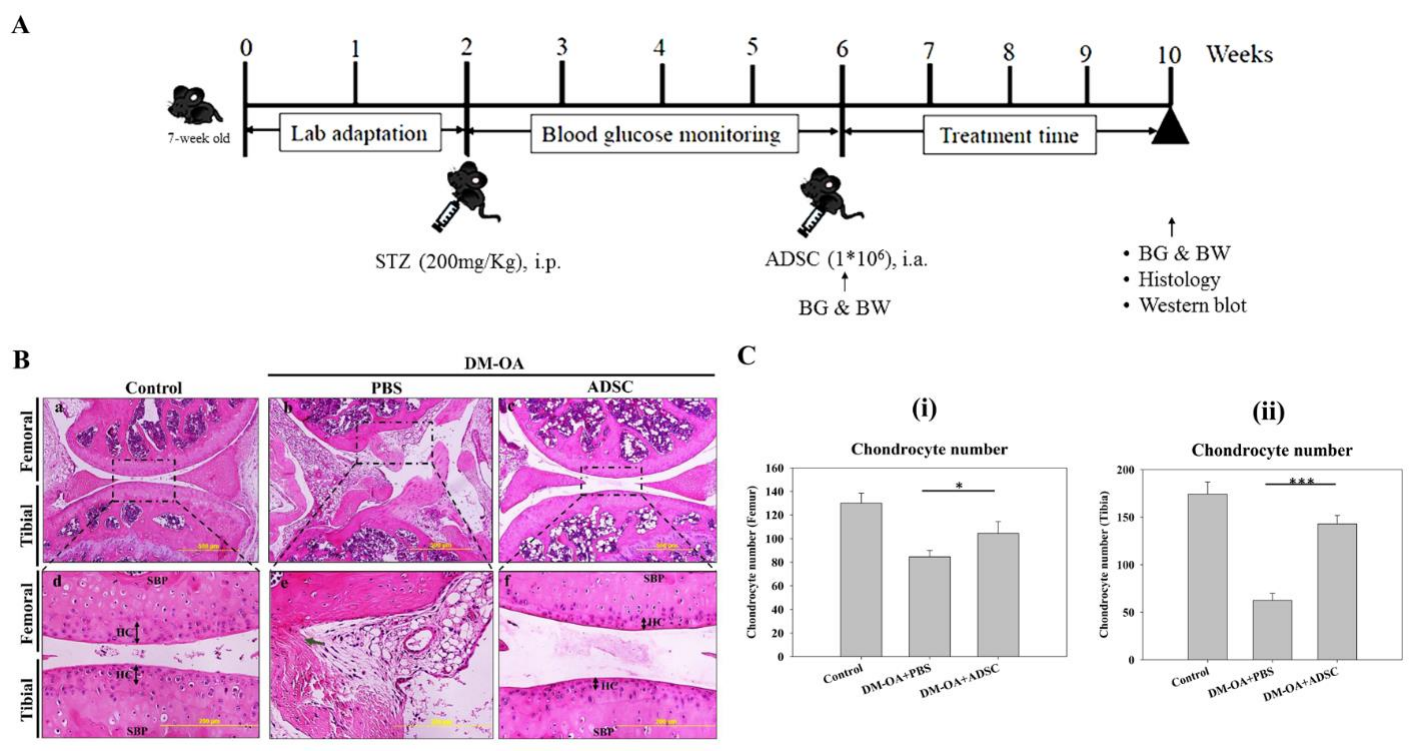
ADSC administration and assessment of histologic structural changes associated with OA (**A**) Experimental protocol for ADSC therapy in STZ-mediated DM-induced OA mice. Following 2 weeks of lab adaptation, mice were rendered diabetic via administration of STZ (200mg/Kg body weight), and then blood glucose level was monitored till 4 weeks. After reaching >300mg/dl, 1×10^6^ ADSC were intra-articularly injected. Thereafter, the improvement in OA status was assessed. (**B**) Representative images of hematoxylin and eosin (H&E) stained sections of mouse knee-joints injected with ADSC or PBS and control after 4 weeks. Rectangular boxed regions indicate the areas shown in higher magnification. The thickness of hyaline cartilage (HC) was indicated by blacked coloured double arrows. SBP represents the subchondral bone plate. Arrows in green, red and yellow colour indicated the cartilaginous lesions in femoral, tibial and meniscal regions. Bar: 500 μm (lower magnification, 10X), 200 μm (higher magnification, 20X). (**C**) Chondrocyte numbers in the femur (i) and tibia (ii) were quantified after 4 weeks of ADSC treatment. BG: blood glucose, BW: body weight. Data are shown as mean±SEM (Control, n=5; DM-OA+PBS, n=6; and DM-OA+ADSC, n=6). *p<0.05 and ** p<0.01.

**Figure 4.**
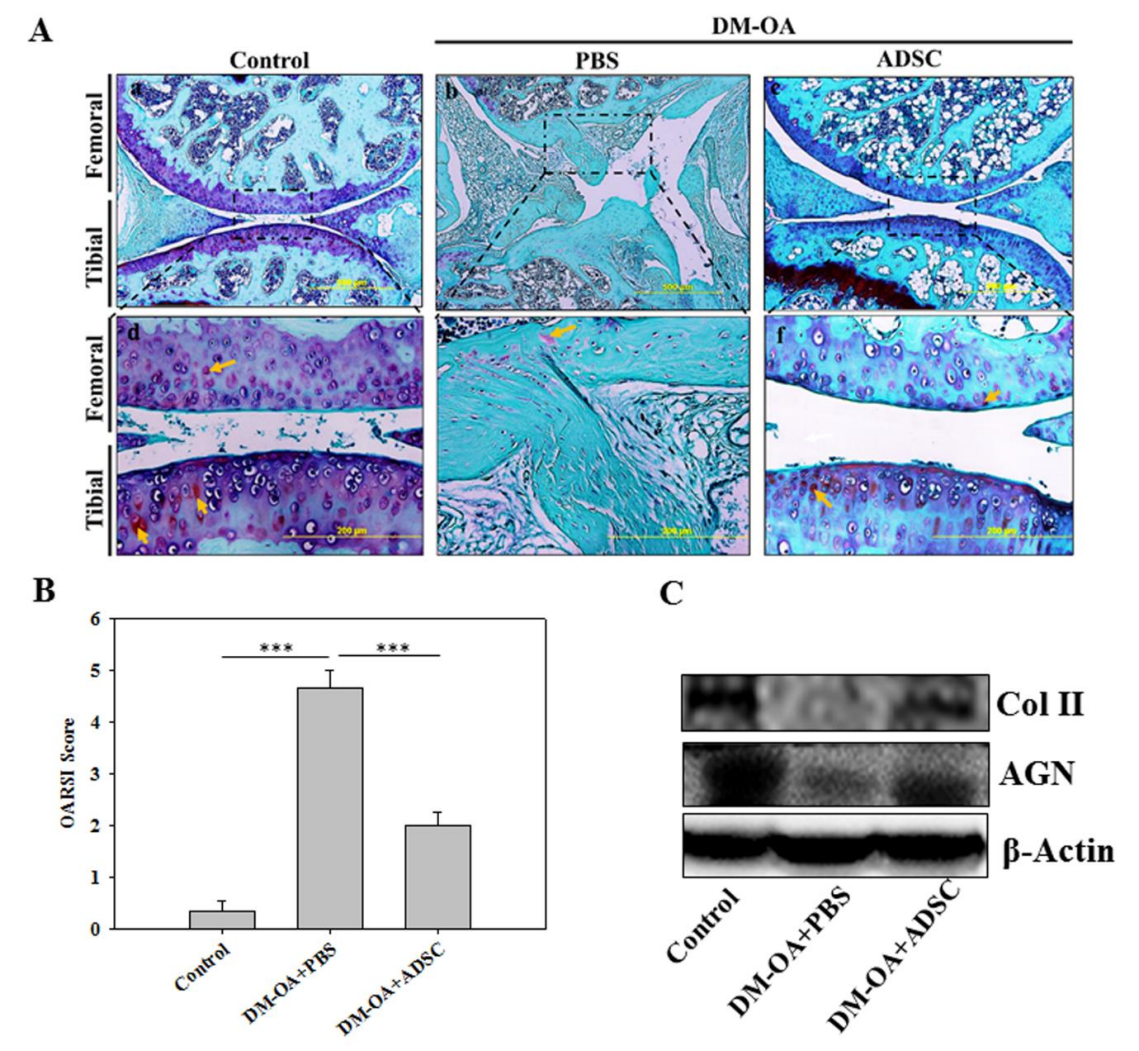
ADSC administration and assessment of proteoglycans (PG) and collagen content in knee-joint of diabetic mice (**A**) Safranin-O/Fast Green staining for knee joint histological evaluation of PG in knee-joints injected with ADSC or PBS and control after 4 weeks. The upper panel of 3 images are at low magnification (500 μm) in which the dotted rectangular boxed regions are represented as their respective images (lower panel) at higher magnification (500 μm). Yellow and white arrows indicate accumulated proteoglycans in articular cartilage and meniscus respectively. (**B**) Severity of articular cartilage degradation was graded using OARSI scoring system. Data are shown as mean±SEM (Control, n=5; DM-OA+PBS, n=6; and DM-OA+ADSC, n=6). *** p<0.001. (**C**) Protein expression of biological markers of chondrogenic origin, including type II collagen and aggrecan (Col II and AGN).

### Regeneration of OA-related impairments in knee-joint of diabetic mice by ADSCs

ADSCs have shown a strong regenerative efficacy in various damaged tissues and organs followed by injuries and disorders [21]. We further addressed the question of whether intra-articularly administered ADSCs could also regenerate OA-associated structural abnormalities in knee-joints of diabetic mice. The administration of ADSCs has been illustrated in schematic form in Fig. 3A. As revealed by H&E staining, after 4 weeks, the PBS-treated DM-OA group exhibited extensive articular cartilage lesions in femoral (green arrow) which was typically characterized by fibrillation of extracellular cartilaginous matrix (ECM), erosion and excavation of unmineralized hyaline cartilage (HC) in superficial and middle layers that gradually extended into the deeper layers (Fig. 3B-e). Furthermore, in this group, hypocellular chondrocytes appeared dying and disoriented in both the femoral and tibial ECM, with no appearance of tidemarks, which signalled an exacerbated OA-like phenotype. A thinning of subchondral bone plate (SBP) was also observed, signifying extended damage to subchondral bone. Notably, severe fibrillations and fraying of meniscal tissues were apparent, indicating susceptibility of fibrochondrocytes similar to hyaline chondrocytes in diabetic microenvironment. The morphology of the control group appeared to be normal, showing no any sign of damage (Fig. 3B, a and d). However, histological analysis revealed that the ADSC-treated DM-OA group joints had a smoother articular surface (Fig. 3B, c and f), though a comparatively thinner HC and SBP compared to control group, indicating attenuation of OA in diabetic milieu. We next quantified chondrocyte number, which were found higher in ADSC treated group compared to PBS-treated DM-OA group in the femur and tibia (Fig. 3C, i and ii). These results strongly indicate that ADSC treatment after the onset of OA can attenuate the noted pathological changes by avoiding the degeneration of the osteochondral unit, including cartilage, meniscus and bone. Interestingly, no amelioration of blood glucose level but a slight increase in body weight was found after 4 weeks treatment of ADSCs in DM-OA mice (Supplemental Fig. 1).

**Figure 5.**
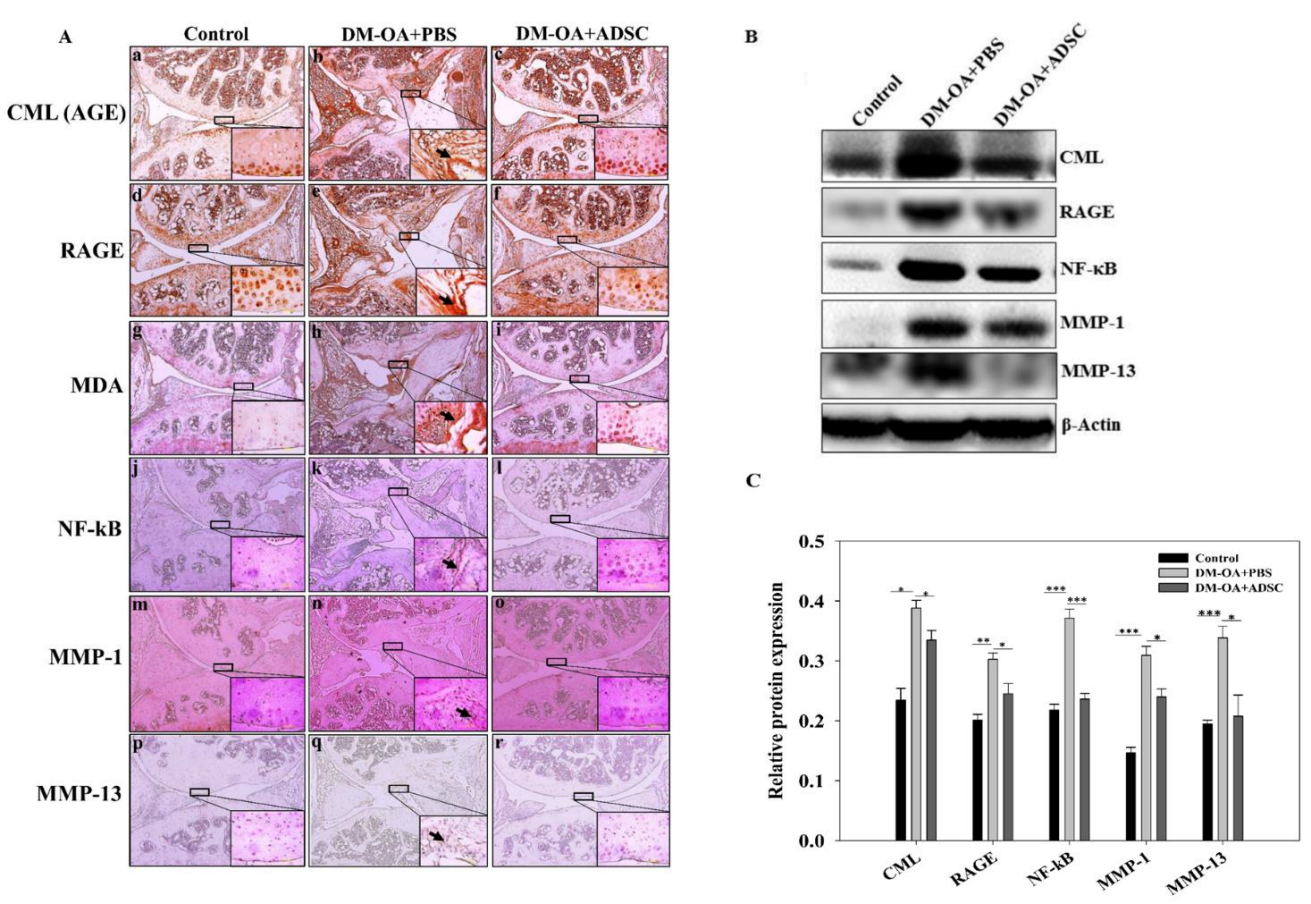
Effect of ADSC administration on expression of AGE-RAGE axis mediated activation of catabolic signalling pathways. (**A**) Immunohistochemistry was performed to assess the expression of CML as AGE (a-c), RAGE (d-f), MDA (g-i), NF-kB (j-l) MMP-1 (m-o) and MMP-13 (p-r) in diabetic knee-joints injected with ADSC or PBS and control after 4 weeks. The rectangular boxed regions in images at lower magnification (500 μm) are represented as their respective images at higher magnification (50 μm). Protein expressions (**B**) and their respective quantification (**C**) of CML, RAGE, NF-κB 65, MMP-1 and MMP-13 in control, DM-OA+PBS or DM-OA+ADSC group. *p<0.05, **p<0.01 and ***p<0.01. CML, carboxymethyl lysine; AGE, advanced glycation end product; RAGE, receptor for advanced glycation end products; NF-κB, nuclear factor kappa B; MMP, matrix metalloproteinase

Aggrecan is a major proteoglycan (PG) present in the articular cartilage, which, in addition to mediating chondrocyte-chondrocyte and chondrocyte-matrix interactions, provides a hydrated gel structure thereby imparting load-bearing properties in the cartilage [22]. Hence, we evaluated whether ADSCs exerted any beneficial effect on proteoglycan and collagen content. Histological examinations of PG-specific safranin o- stained knee-joint sections in the control group demonstrated normal architecture of articular cartilage and meniscus with intense red signals (indicated by yellow color arrowhead) (Fig. 4A, a and d). In contrast, the PBS-treated DM-OA group revealed a massive PG loss, represented by absence of safranin o staining and severe fragmentation and fibrillation of articular cartilage and meniscal tissues (Fig. 4A, b and e), representing an OA-like characteristics. However, the staining of tissue sections appeared to be increased in these regions of ADSC-treated DM-OA group mice at 4 weeks (Fig. 4A, c and f). We further conducted a semi-quantitative histopathologic grading of Safranin-O/Fast Green-stained knee joint cartilage using a murine scoring system established by Osteoarthritis Research Society International (OARSI) initiative [20]. The PBS-treated DM-OA group scored significantly higher than control mice, indicating severe osteoarthritic changes in knee-joint compared to control (Fig. 4B). Consistent with these findings, the protein expression of chondrogenic markers (Col II and AGN) were also extremely reduced in PBS-treated DM-OA group compared to control group; however, ADSC reversed their expression level (Fig. 4C) implying enhanced collagen and PG content. These results suggest that the diabetic microenvironment adversely affected cartilage biology, particularly the net loss of collagen and PG aggregates, which were mitigated by intervention of ADSCs.

### ADSCs-mediated inhibition of expression of carboxymethyl lysine (CML), an advanced glycation end product (AGE), and their receptors (RAGE) in diabetic knee-joint

The synthesis and accumulation of AGEs have been reported to progress at an accelerated rate under diabetes [23]. We speculated that accumulated CML through binding on RAGE, mediates signalling, which is critical for regulating cartilage homoeostasis [24]. Hence, to investigate the relationship between CML and the osteoarthritic progression in diabetic mice, CML and RAGE, a specific receptor of CML, in the cartilaginous lesions were detected by IHC and Western blot analysis. Strikingly, compared to control group (Fig. 5A-a), an intense staining of CML in the knee-joint tissues, including articular cartilage and meniscus, was demonstrated in the PBS-treated DM-OA group (Fig. 5A-b), which was found to be attenuated by ADSC intervention (Fig. 5A-c). Subsequently, we investigated whether RAGE was expressed in knee-joint tissues; the RAGE immunostaining was found elevated in PBS-treated DM-OA group (Fig. 5A-e) as compared to control group (Fig. 5A-d). Specifically, the staining was more prominent in articular cartilage and meniscal regions in the PBS-treated DM-OA. However, declined immunostaining to a feeble level was exhibited in the ADSC-treated group (Fig. 5A-f). These results were further confirmed via western blot analysis and quantified (Fig. 5B & C, CML and RAGE panel). Overall, the above data were indicative of ADSC-mediated inhibition of activated AGE-RAGE axis.

### Suppression of oxidative stress in knee-joint of diabetic mice by ADSC

Since the engagement of RAGE with CML evokes oxidative stress via hyper-production of reactive oxygen species (ROS) [25, 26], we conducted IHC staining to detect the level of malondialdehyde (MDA), an end product of lipid peroxidation and biomarker of oxidative stress in knee-joint. The results of IHC staining showed a very light immunopositive staining of MDA in sections of the control group (Fig. 5A-g), whereas the PBS-treated DM-OA group demonstrated a marked immunopositive staining (Fig. 5A-h). However, in the ADSC-treated group, a reduced MDA-positive staining was revealed (Fig. 5A-i). The IHC staining analysis showed that MDA upregulation was strongly associated with elevated oxidative stress in DM-OA group, which was supressed by intervention of ADSCs. Thus, high MDA expression might serve as a risk marker for oxidative stress thereby contributing in progression of osteoarthritic characteristics.

### Reduction of over-expression and activation status of NF-κB in knee-joint of diabetic mice by ADSCs

NF-κB expression is an indicator of apoptosis and inflammation in the knee-joint, and was assessed by IHC staining in control, PBS or ADSC-treated DM-OA group. IHC revealed that NF-κB staining was intense in the PBS-treated DM-OA group (Fig. 5A-k), compared to the control group (Fig. 5A-j), while an evident reduction in staining was noted in ADSC-treated DM-OA group (Fig. 5A-l), indicating inhibition of activated NF-κB. Consistent with these findings, western blot results and their quantification revealed that the protein expression of NF-κB was also increased in DM-OA group relative to control group and found to be mitigated in ADSC treated DM-OA group (Fig. 5B & C, NF-κB panel).

### Efficacy of ADSC treatment on matrix metallo-proteinase (MMP) 1 and 13 in knee-joint of diabetic mice

NF-κB has been attributed to activation of MMP promoters, and the induction of degradative MMPs, particularly, MMP-1 and 13 which are collagenases in nature, plays a pivotal role in the destruction of cartilage, thereby leading to development of OA [27]. Therefore, to corroborate the role of NF-κB signalling in inducing the production of catabolic proteases MMP-1 and 13 in the cartilage matrix, we conducted IHC staining, which revealed increased expression of MMP-1 and 13 in the PBS-treated DM-OA group (Fig. 5A, n and q panel) compared to the control (Fig. 5A, n and q). The decreased expression of MMP-1 and MMP-13 was evidenced in ADSC-treated DM-OA group (Fig. 5A, n and q). These results were further validated by western blot analysis and quantified (Fig. 5B& C, MMP-1 & 13 panel). These findings indicate that ADSCs protect cartilage against degradation partly by inhibiting MMP-1 and MMP-13.

**Figure 6.**
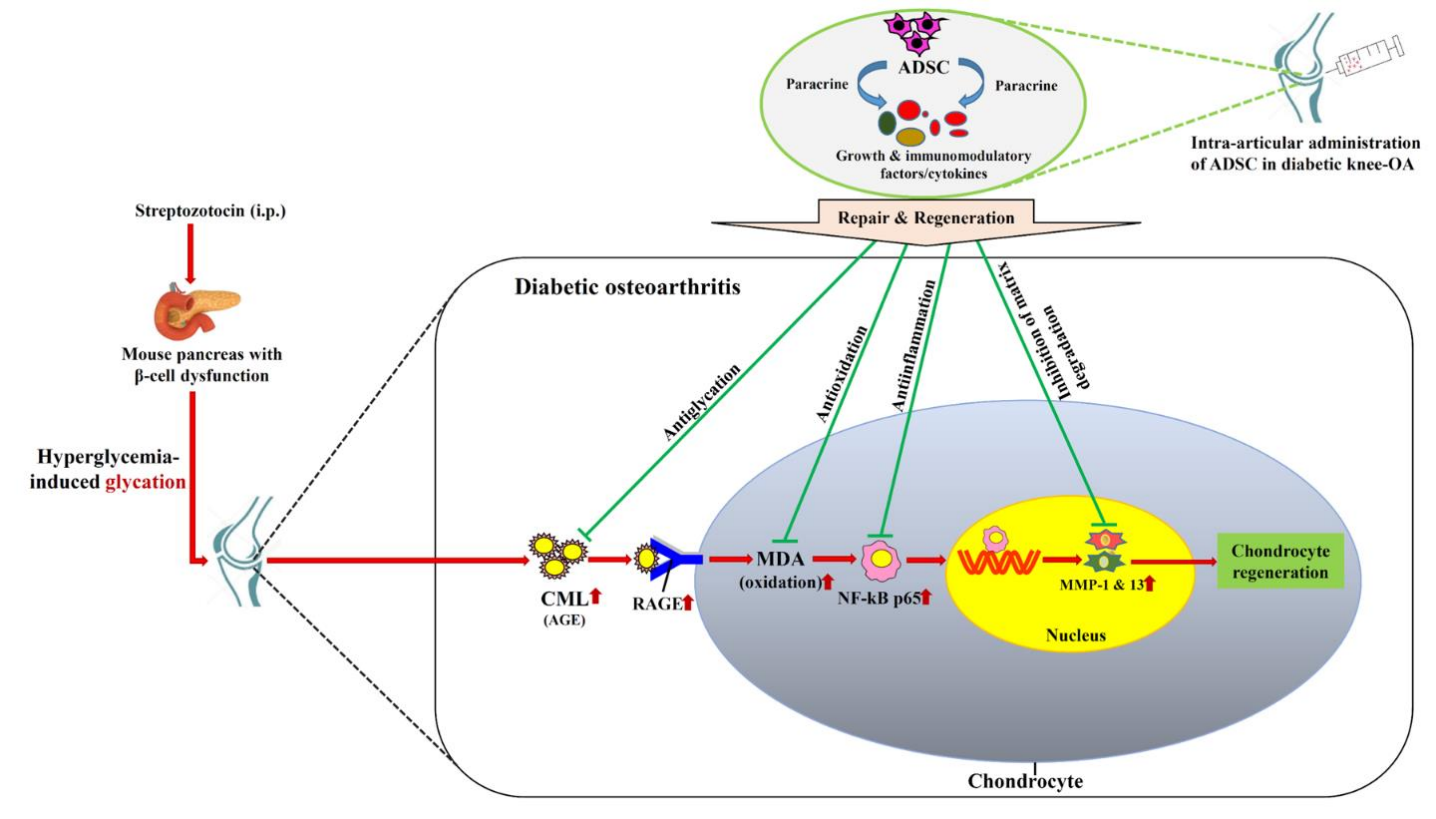
Schematic of possible mechanistic insight of therapeutic action by ADSC in osteoarthritic knee-joint of diabetic mice.

## DISCUSSION

All forms of diabetic hyperglycemia, leads to various debilitating pathologies [28]. However, with soaring rates of the prevalence of DM worldwide [29, 30], the possible detrimental impact of hyperglycemia on cartilage health leading to osteoarthritic characteristics is a major concern. This study demonstrated that DM potentiates the initiation and progression of OA in mouse knee joints by disrupting the articular cartilage and meniscus, and also investigated whether ADSCs could exert therapeutic activity in DM-induced OA through regenerative and anti-inflammatory mechanisms. In previous studies, the regenerative effects of ADSC have been demonstrated in various tissue including periodontal and nerve tissues [31, 32]. Since intra-articularly administered CM-DiL labelled ASDCs were observed in the knee-joint, the therapeutic effects of ADSCs on OA in diabetic microenvironment appeared attributable to various paracrine factors. As revealed by H&E staining, the deteriorating morphological and histological changes of articular tissues, including cartilage, meniscus and subchondral bone, were indicative hallmarks of osteoarthritis showing compromised knee-joints, which were restored by ADSC treatment (Fig. 3B). Aggrecan is the multi-faceted PG contained in the articular cartilage which imparts stability to ECM and form the hydrated pressure-resistant gel which lubricates joints [22]. The massive degradation of PG in the diabetic group further supported the hypothesis that diabetic microenvironment participates in the initiation and progression of OA. Further, the enzyme, glucosaminyl N-deacetylase is known to catalyse the first step in the integration of sulphate groups (sulphation) to the glycosaminoglycan chain in aggrecan, which is a pivotal step in determining overall structure and charge density of PG [33]. Since this study revealed noticeable changes in articular cartilage and meniscus, which appeared as degraded PG in diabetic group, it indicated complete loss of viscoelastic biomechanical property, a prominent characteristic of OA. The reduced PG content might be attributed to decreased sulfate groups during impaired deacetylation in hyperglycemic microenvironment. This result is in accord with a previous study demonstrating suppressed N-deacetylase activity in hepatocytes of diabetic rats [34]. However, the intra-articularly introduced ADSCs in the DM-OA group demonstrated an increased PG content (Fig. 4A and C), which might be ascribed to insulin growth factor-1 (IGF-1) mediated stimulation of PG synthesis by chondrocytes [35]. The anti-apoptotic activity of IGF-1 as a secreted bioactive soluble factor by MSCs, specifically by ADSCs, have also been reported [36]. In another study, ADSC secreted IGF-1 improved proliferation and viability of myostatin treated myoblasts, indicating their potential therapeutic ability [37].

Under a hyperglycemic environment, non-enzymatically formed AGEs are considered as a fatal mediator in the diabetic atherosclerotic pathology [38], and may also account for musculoskeletal dysfunction, including OA. Notably, the formation and accumulation of AGEs progress at an accelerated rate under diabetic conditions [39]. The AGE formation on ECM not only interferes with matrix-matrix interactions, but also with matrix-cell interactions. Furthermore, the growing *in vitro* evidence shows that AGEs after interacting with their signal-transducing receptor (RAGE) in chondrocytes [40, 41], evoke oxidative stress and subsequently elicit inflammatory responses. The mice employed in this study were rendered diabetic by the administration of STZ, which selectively deteriorates the β-cells of the pancreatic islets, thereby resulting in hyperglycemia and the subsequent formation of endogenous AGEs. In this concord, the observed intense signals of CML and RAGE in the articular cartilage and meniscal regions indicate their cross-linking with collagenous matrices, which may serve as a link between CML deposition and pathogenesis of OA. Our results also revealed that ADSC treatment effectively inhibited the increased CML and RAGE levels, suggesting an anti-glycation activity of ADSC (Fig. 5A & B, CML and RAGE panel). The AGEs accumulation exerts oxidative stress and inflammation in many tissues, including skin and kidney [12, 42]. Hence to confirm whether ADSCs exerted a protective effect in diabetic knee-joint by anti-oxidative action, we assessed the expression levels of MDA, an indicator of lipid peroxidation. Expectedly, MDA levels were dramatically increased in PBS treated DM-OA group; however, ADSCs treatment decreased the MDA levels (Fig. 5A, MDA panel). The oxidative stress also activates NF-κB [43], an essential transcription factor and major upstream mediator of many pro-inflammatory molecules associated with osteoarthritic synovium [44-46]. Furthermore, the NF-κB signalling pathway participates in the etiopathogenesis of OA [47]. In previous reports, high glucose induced NF-κB activity in human glomerular endothelial cells, vascular smooth muscle cells and mononuclear cells has been documented [48, 49], indicating the possibility of exacerbated and sustained NF-κB activity in chondrocytes. In concord with these studies, we found that ADSCs suppressed DM-induced NF-κB activation, indicating the repression of inflammatory response (Fig. 5A and B, NF-κB panel).

The key characteristic features of OA chondrocytes include exacerbated and sustained NF-κB activation and production of MMPs [50], which may compromise the integrity of the ECM of articular cartilage. Interestingly, glucose can modulate the production, expression, and activity of MMPs in specific cell lines [51]. These previous reports indicate that diabetic microenvironment may enhance NF-κB activity and synthesis of MMPs, which alter the biomechanical characteristics of ECM. Of note, MMPs are a family of ECM-degrading enzymes, which catalyse both collagen and proteoglycan degradation including collagenases (MMP-1 and 13), the expression of which are enhanced in high glucose concentration [52, 53]. MMP-13 has been reported as a highly expressed proteinase in OA, which can degrade both type II collagen and aggrecan [54]. Therefore, we hypothesized that DM-induced OA is related to the up-regulation of MMP-1 and 13 in the knee-joint [55]. In agreement with the above reports, our data revealed the elevated levels of MMP-1 and 13 in DM-OA group which were highly suppressed after the ADSC intervention (Fig. 5A & B MMP-1 & 13 panels). Taken together, Diabetic OA is caused principally through hyperglycemia-mediated metabolic diseases, which imparts functional changes in knee joint. Besides, the factors such as oxidative stress, inflammation, deterioration of extracellular matrices through activated NF-κB signaling have been documented to explain the mechanism of histopathological deformities in diabetic OA knee. These beneficial effects are in accordance with previous studies showing ability of ADSC toward differentiation to tissue specific cells, reducing inflammation through modulating the immune system [56-58], stimulation of angiogenesis [59, 60], and inducing both cell migration as well as proliferation [61, 62], differentiation and extracellular matrix formation [63, 64]. Therefore, these functional benefits observed after ADSC transplantation in experimental models of diabetic OA might be related to the secreted paracrine/trophic factors [65]. However, though ADSC demonstrated curative effects in the treatment diabetic OA, further studies are needed to comprehensively elucidate the cell-signalling pathways and molecular mechanisms underlying these findings. As this study employed type 1 DM mice model, alternative investigation in type 2 DM-induced OA will broaden the concepts about comparative hyperglycemic effects on OA. As the pathophysiological state of diabetic OA is induced by various disorders, the pleiotropic mode of actions by ADSC therapy seems highly effective in treatment of OA in hyperglycemic microenvironment.

Conclusively, our results corroborated diabetic complication of OA, and deduced the possible therapeutic mechanism associated with ADSC therapy in comorbid condition of diabetic OA. During repair and regeneration in diabetic OA knee-joint, the administered ADSCs halted the osteoarthritic characteristics in spite of undiminished hyperglycemia in diabetic mice. Taken together, our findings suggested that during *in vivo* regeneration and repair, ADSC inhibited glycation-mediated inflammatory cascade, particularly through anti-glycation, anti-oxidation and anti-inflammatory activities in osteoarthritic knee-joint in hyperglycemic milieu (Fig. 6).

## Supplemetary Material

The Supplemenatry material for this article can be found online at: www.aginganddisease.org/EN/10.14336/AD.2018.0616


