# Supplementary material for: Adipose-derived Stem Cells Attenuates Diabetic Osteoarthritis via Inhibition of Glycation-mediated Inflammatory Cascade
Source: Aging Dis. 2019 Jun 1;10(3):483–96. doi: 10.14336/AD.2018.0616 (PMC6538220; doi:10.14336/AD.2018.0616)
Supplement: Supplementary file 1 [file AD-10-3-483-s.pdf]

SUPPLEMENTARY DATA

Adipose-derived Stem Cells Attenuates Diabetic Osteoarthritis via Inhibition of Glycation-mediated Inflammatory Cascade

Navneet Kumar Dubey[1,2], Hong-Jian Wei[2,3,4], Sung-Hsun Yu[2], David F. Williams[5], Joseph R. Wang[6], Yue-Hua Deng[7], Feng-Chou Tsai[8], Peter D. Wang[4,9], Win-Ping Deng[2,4,10*]

[1]Graduate Institute of Biomedical Materials and Tissue Engineering, College of Biomedical Engineering, Taipei Medical University, Taipei, Taiwan
[2]Stem Cell Research Center, College of Oral Medicine, Taipei Medical University, Taipei, Taiwan
[3]School of Dental Technology, College of Oral Medicine, Taipei Medical University, Taipei, Taiwan
[4]School of Dentistry, College of Oral Medicine, Taipei Medical University, Taipei, Taiwan
[5]Wake Forest Institute of Regenerative Medicine, Winston-Salem, NC, USA
[6]Department of Periodontics, College of Dental Medicine, Columbia University, New York, USA
[7]Department of Life Science, Fu Jen Catholic University, New Taipei City, Taiwan
[8]Stem Cell Research Center, Cosmetic Clinic Group, Taipei, Taiwan
[9]Department of Dentistry, Taipei Medical University Hospital, Taipei, Taiwan
[10]Graduate Institute of Basic Medicine, Fu Jen Catholic University, New Taipei City, Taiwan

© 2018. Chen W et al. Published online at http://www.aginganddisease.org/EN/10.14336/AD.2018.0615

SUPPLEMENTARY DATA

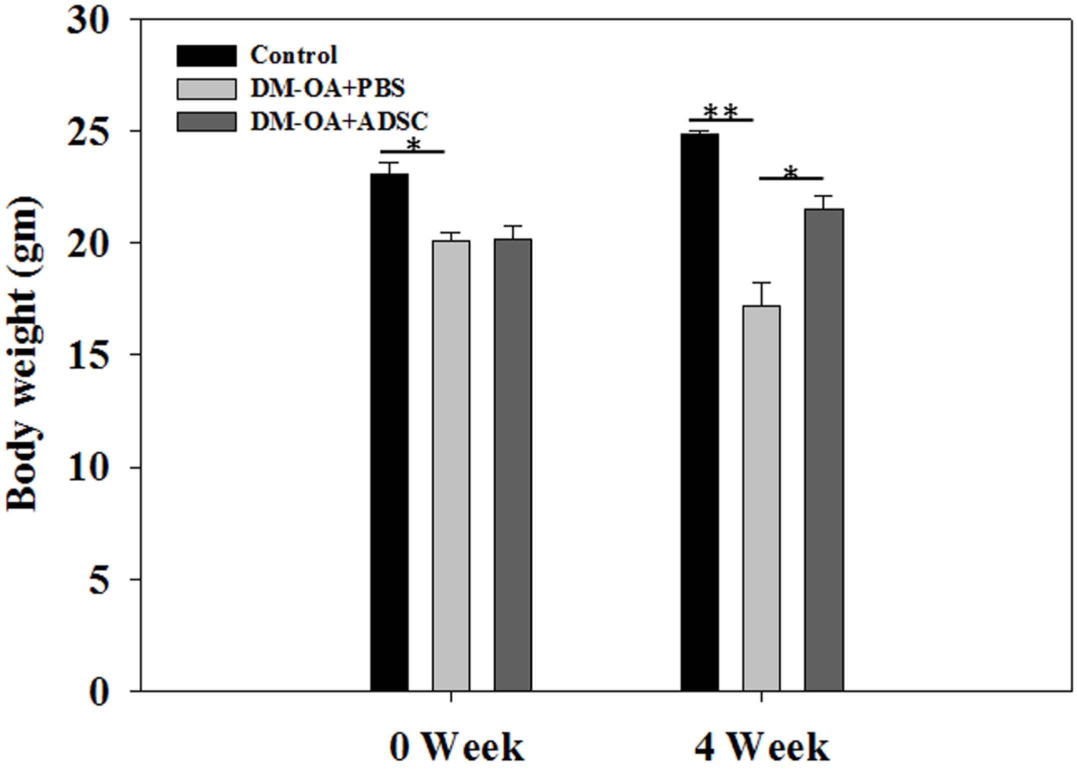

Supplementary Figure 1. ADSC administration and evaluation of body weight in knee-joint of diabetic mice. (Control, n = 5; DM-OA+PBS, n=6; and DM-OA+ADSC, n=6). *p < 0.05 and ** p<0.01.

© 2018. Chen W et al. Published online at http://www.aginganddisease.org/EN/10.14336/AD.2018.0615
